# Audiology at the Intersection of Labor, Revenue, and Professional Taxonomy

**DOI:** 10.1055/s-0046-1825787

**Published:** 2026-10-06

**Authors:** Victor Bray

**Affiliations:** Osborne Audiology Department, College of Nursing and Health Professions, Drexel University, Elkins Park, Pennsylvania, United States

**Keywords:** professional taxonomy, labor economics, healthcare reimbursement, audiology practice, revenue generation

## Abstract

A healthcare profession's economic position is determined not only by its educational preparation or clinical scope, but also by how its services are classified, reimbursed, and translated into professional labor demand. This article examines audiology through the integrated framework of professional taxonomy, revenue generation, and labor economics to explain the structural factors underlying the profession's current economic position within the U.S. healthcare system. Drawing on principles of labor economics and evidence synthesized from companion analyses of workforce supply and professional earnings, the manuscript characterizes how regulatory classification, reimbursement authority, and prevailing revenue models interact to shape the valuation and deployment of audiologic labor. The analysis argues that audiology occupies a hybrid position spanning diagnostic, therapeutic, and product-linked models without achieving full economic recognition within any of them. This structural misalignment limits independent billing authority, constrains pricing power, weakens the relationship between clinical expertise and professional revenue, and contributes to compressed earnings, restricted workforce growth, and limited scalability of service delivery. Understanding these interdependent structural relationships provides an explanatory framework for interpreting audiology's workforce and economic challenges and establishes a foundation for evaluating future policy, reimbursement, and practice model reforms intended to strengthen the profession's long-term sustainability.

## Introduction

In healthcare, the economic position of a profession is shaped by more than the training it requires or the patients it serves. It is shaped by how the profession is classified within regulatory and reimbursement systems, how that classification translates into billable revenue, and how revenue structures in turn determine how professional labor is deployed and compensated. Taxonomy, revenue generation, and labor markets are not independent domains—they operate as an interdependent system, and misalignment among them produces predictable, durable economic consequences.

Professions that have achieved alignment across these domains demonstrate how the system can function effectively. Nurse practitioners and physician assistants, for example, gained independent prescribing authority and expanded reimbursement recognition—changes in taxonomy that opened new revenue pathways and supported wage growth consistent with their training investment. 1 Physical therapists achieved direct access legislation and doctoral-level entry requirements in tandem with reimbursement structures that support longitudinal, visit-based care. In each case, taxonomy, revenue model, and labor deployment evolved together, reinforcing a professional economic position consistent with training and clinical scope. 2

Audiology presents a contrasting case. Despite doctoral-level preparation and a clinical scope that spans diagnostic evaluation, rehabilitation, and elements of chronic care management, the profession does not map cleanly onto any single established revenue model in healthcare. It is not primarily a diagnostician-centered profession, where evaluation leads to reimbursable medical management. It is not primarily a therapy-based profession, where revenue is tied to time, frequency, and longitudinal care pathways. And while a substantial portion of audiologic care has historically been delivered within technology-linked or product-bundled arrangements, audiology lacks the practice ownership patterns and pricing authority that allow professions such as optometry and veterinary medicine to convert product-linked care into scalable professional revenue.

The result is that audiology occupies a hybrid position at the intersection of medical diagnostics, prescribed technology, and time-based professional services—without full economic recognition in any of these domains. Reimbursement pathways are narrowly defined, cognitive clinical labor is systematically under-recognized, and professional revenue is frequently mediated through external entities including referral requirements, manufacturer-linked distribution channels, and benefit design structures. Increases in population-level demand for hearing care do not reliably translate into proportional increases in demand for independently billable audiologic services. This structural positioning—not insufficient training, not inadequate clinical scope, not lack of patient need—is the primary driver of audiology's economic constraints.

The purpose of this manuscript is to examine that positioning through the integrated lens of professional taxonomy, revenue generation, and labor economics. Drawing on established economic principles and on evidence presented in three companion articles within this special issue–one examining workforce supply (see Audiology Workforce Supply and Population Demand: Trends, Benchmarks, and Implications), one addressing audiology wages (see Economics of Audiology Wages: Benchmarking a Doctoral Health Profession Within U.S. Healthcare), and one evaluating educational investment (see Shaping the Future of Audiology: Current and Evolving Landscape of Audiology Education)–this analysis characterizes how current classification structures and revenue pathways shape the deployment and valuation of audiologic labor. In doing so, it moves beyond description of outcomes to examine the institutional architecture that produces them. This analysis is intentionally structural and diagnostic: it does not evaluate whether specific interventions are likely to produce alignment, assess the feasibility of proposed reforms, or prescribe policy solutions. Those evaluative and translational functions are the work of companion analyses in this special issue. The contribution of this manuscript is the structural account itself—a characterization of how taxonomy, revenue, and labor operate as an interdependent system—which is a prerequisite for accurately interpreting the profession's current challenges and for framing the evaluative questions that follow.

## Professional Taxonomy as a Structural Constraint

In U.S. healthcare, professional taxonomy operates through two primary classification systems that together determine how a profession is recognized, how its services are reimbursed, and what economic authority it holds within the healthcare system. The Bureau of Labor Statistics (BLS) Standard Occupational Classification (SOC) system defines how professions are categorized for workforce and labor market purposes, establishing the degree requirements and occupational boundaries used in national wage reporting. 3 The Centers for Medicare and Medicaid Services (CMS) classification system determines how providers are recognized within federal reimbursement structures—specifically, whether a profession is designated as a physician, a limited license practitioner, or a supplier of services. 4 These classifications are not merely descriptive. They are economically determinative: they establish which reimbursement pathways are accessible, which billing codes may be used, and whether clinical judgment can be independently translated into billable activity.

Audiology's classification within the CMS system imposes specific and consequential constraints. Under Section 1861 of the Social Security Act, audiologists are designated primarily as suppliers of diagnostic services rather than as limited license practitioners or limited license physicians. 4 This designation restricts independent billing authority, reinforces referral-dependent care models, and excludes audiologists from utilizing evaluation and management (E&M) codes (CPT 99202–99215)—the billing mechanism through which diagnostician-centered professions capture the value of clinical assessment, interpretive reasoning, and care planning. Because audiologists routinely perform each of these functions—determining appropriate testing protocols, interpreting diagnostic findings, and developing management plans—the E&M exclusion represents a structural gap between the cognitive labor performed and the reimbursement mechanisms available to recognize it. The result is that a substantial portion of audiologic clinical activity remains economically unrecognized within the primary federal payment framework.

These classification constraints are consistent with functioning as an upstream constraint on audiology's economic position. They shape which services are viable, how care is structured, and whether professional labor can be independently valued—and they do so prior to and independently of any individual clinician's training, competence, or productivity. Taxonomy, in this sense, does not merely reflect professional status; it actively constructs the economic boundaries within which professional labor operates. For audiology, those boundaries have remained misaligned with the profession's clinical scope and training requirements, establishing the structural conditions that the sections that follow will examine in terms of revenue generation and labor market outcomes.

## Revenue Generation Models in Healthcare

Healthcare professions generate revenue through a limited number of dominant models, each defined by how clinical activity is translated into reimbursable services. Broadly, these models can be categorized into three types: diagnostician-centered, therapy-based, and product-linked. Understanding where audiology sits relative to these models—and why it does not fit cleanly within any one of them—is essential to understanding the profession's structural economic constraints.

Diagnostician-centered models, characteristic of physicians, nurse practitioners, and physician assistants, generate revenue through E&M services, procedural interventions, and ongoing medical decision-making. In these models, diagnostic activity leads directly to reimbursable treatment pathways, and clinical judgment is consistently recognized as independently billable labor. Therapy-based models, characteristic of physical therapy, occupational therapy, and speech-language pathology, generate revenue through time-based or visit-based services structured longitudinally, with reimbursement tied to frequency, duration, and progression of treatment. These models support predictable revenue streams and facilitate scalable delivery through standardized care pathways. Product-linked models generate revenue through the provision of medical devices or technology, often accompanied by associated professional services. In optometry and veterinary medicine—the professions most structurally analogous to audiology in this respect—product-linked care is augmented through practice ownership, control of service pricing, and integration of clinical and retail components, allowing professional revenue to scale alongside product transactions.

Audiology does not map cleanly onto any of these models. It does not generate revenue primarily through diagnosis leading to insurance-backed procedures established on medical necessity, as physicians, dentists, and podiatrists do. It does not generate revenue tied to time, visits, or frequency of care, as chiropractors, physical therapists, occupational therapists, and speech-language pathologists do. And while audiology shares structural features with optometry and veterinary medicine—clinical examination authorizing prescribed products—it has not consistently achieved the practice ownership patterns and pricing authority that allow those professions to convert product-linked care into scalable professional revenue. Instead, audiology occupies a hybrid position combining medical diagnostics with limited diagnostic reimbursement, high-cost prescribed technology competing within a consumer electronics market, and time-based professional services frequently delivered as bundled or complementary components of a product transaction. This positioning—simultaneously medical, retail, and therapeutic, yet not fully recognized within any of those frameworks—is the structural condition underlying the profession's constrained economic authority.

### Reimbursement Structure and Revenue Capture

A critical component of revenue generation in healthcare is the mechanism through which clinical labor is translated into reimbursable services. Within Medicare and many private payer systems, this operates primarily through two pathways: procedure-based valuation expressed through relative value units (RVUs) and E&M services, which capture clinical judgment, diagnostic reasoning, and care planning.

In audiology, reimbursement is predominantly anchored in procedure-based valuation. Routine audiologic services are assigned RVUs that emphasize the technical components of care, with limited recognition of the cognitive labor associated with clinical decision-making. As established in the taxonomy section above, audiologists are currently excluded from utilizing E&M codes (CPT 99202–99215) under CMS policy—the billing mechanism that diagnostician-centered professions use to capture the value of assessment, interpretation, and care planning. 4 Because audiologists routinely determine testing protocols, interpret diagnostic findings, and develop and implement management plans, this exclusion represents a structural gap between the scope of clinical judgment exercised and the reimbursement mechanisms available to recognize it. A substantial portion of audiologic clinical activity—particularly activities involving synthesis, interpretation, and care coordination—remains economically uncaptured within existing payment structures.

The economic consequences of this exclusion are most acute in physician-owned and physician-supervised practice settings, where the E&M gap becomes an active extraction mechanism rather than a passive limitation. In these arrangements, audiologists conduct the clinical encounter in full—performing the evaluation, interpreting findings, and formulating the management plan—while the billing is captured under the supervising physician's license at E&M reimbursement rates that the audiologist cannot independently access. 4 The audiologist provides the labor; the physician, as the credentialed owner of the billing mechanism, captures the surplus that labor generates. The arrangement is structurally analogous to sharecropping: the professional who works the land does not own it, and the economic value of that work flows to the party who controls the title—in this case, the CPT authorization. That CMS already requires audiologic services in physician offices to be billed under the audiologist's own NPI—while simultaneously denying the E&M authority that would make that billing economically meaningful—captures the precise nature of the constraint: recognition without reimbursement.

The downstream consequence is a hybrid revenue structure in which professional labor is only partially recognized through formal reimbursement mechanisms. In many practice settings, clinical services are embedded within or effectively subsidized by technology transactions rather than independently priced as professional care. 5 Unlike optometry and veterinary medicine, where practice ownership and integrated service-product pricing allow professionals to capture revenue across both clinical and retail components, independent ownership and pricing control are less consistently realized in audiology. 6 7 This limits the profession's ability to convert clinical expertise into scalable, consistently valued revenue—even in the practice settings most structurally positioned to do so.

### Revenue Mediation

Beyond classification constraints and reimbursement gaps, audiology revenue is frequently mediated through external entities rather than generated directly by professional labor. Referral requirements position audiologic care as downstream of physician authorization, limiting direct patient access and constraining the profession's ability to independently establish and capture service value. Manufacturer-linked distribution channels and third-party benefit structures further influence how services are accessed, priced, and delivered—in some arrangements, effectively determining which products are dispensed, at what price, and through which providers. 5

The economic consequence of this mediation is a decoupling of population-level demand from institutional demand for audiologic services. Growing prevalence of hearing loss, expanding evidence for the health consequences of untreated hearing impairment, and increased public awareness of hearing technology do not automatically translate into increased demand for independently billed audiologic care. When revenue pathways are intermediated by entities that control access, pricing, or distribution, the economic value generated by clinical expertise is partially captured upstream—by referral sources, device manufacturers, or benefit administrators—rather than by the profession itself. This decoupling weakens the direct relationship between clinical labor and revenue generation and establishes the conditions under which labor market outcomes, examined in the section that follows, are predictably constrained.

A structural feature that amplifies these mediation effects is the vertical integration of major hearing aid manufacturers into the entities that historically functioned as separate market participants. Leading manufacturers have acquired or established ownership stakes in third-party administrator networks that determine benefit design and reimbursement rates, retail clinic chains through which hearing technology is dispensed, and direct-to-consumer distribution platforms that route patients outside traditional audiologic care pathways entirely. 8 This consolidation concentrates control over product access, coverage determination, and service delivery within entities whose economic incentives are organized around device transactions rather than professional service valuation. The consequence for independent audiologic practice is a market structure in which the profession competes for patients, negotiates reimbursement rates, and attempts to establish service pricing against entities that simultaneously control upstream supply, downstream distribution, and the benefit structures that govern patient access. Unlike optometry—where comparable retail integration has occurred, but independent practice has been partially insulated by preserved E&M billing authority and direct insurance reimbursement for examination services—audiology's weaker reimbursement foundation offers limited structural protection against the economic gravity of vertically integrated hearing care systems. The result is not simply that revenue is captured upstream, but that the architecture of the market is organized in ways that structurally subordinate independent professional services to product-centered transactions.

## Labor Market Implications

Labor economics predicts that when revenue pathways are constrained or fragmented, demand for professional labor becomes similarly constrained—regardless of underlying patient need or population prevalence. This prediction follows directly from the human capital model: professions whose clinical labor cannot be reliably translated into independently valued, reimbursable activity will not experience wage growth commensurate with their educational investment, even as training requirements expand. 9 The structural constraints on audiology's taxonomy and revenue generation documented in the preceding sections are not, therefore, incidental to the profession's labor market outcomes. The evidence presented here is consistent with these constraints functioning as primary drivers of those outcomes—operating prior to and independently of individual clinician training, competence, or productivity.

The wage data presented in Table 1 —and in the companion analyses of this special issue 10 —reflect these structural conditions with consistency. Despite doctoral-level entry requirements established in 2007, audiology wages have not separated meaningfully from those of speech-language pathology—a master's-level profession with an adjacent clinical scope—with mean annual wage differences averaging $256 over a 26-year period and regression slope differences of only $91 per year following the master's degree to doctoral-degree transition.

**Table 1:** Presents mean annual wages and wage growth for 34 healthcare occupations grouped by degree level, illustrating the labor market positioning that results from these structural conditions

Healthcare professions by degree	BLS SOC	Mean annual wage (2022–2024)	Wage growth (2012–2014 to 2022–2024)
Associate's degree			
Radiation therapists	29–1124	$104,527	28%
Nuclear medicine technologists	29–2033	$94,793	32%
Dental hygienists	29–1292	$89,547	25%
Diagnostic medical sonographers	29–2032	$88,660	32%
Magnetic resonance imaging technologists	29–2035	$86,383	30%
Respiratory therapists	29–1126	$79,637	38%
Radiologic technologists and technicians	29–2034	$75,083	32%
Health information technology and medical registrars	29–9021 a	$69,650	NA
Cardiovascular technologists and technicians	29–2031	$69,550	29%
Veterinary technologists and technicians	29–2056	$43,697	37%
Bachelor's degree			
Medical dosimetrists	29–2036 a	$134,117	NA
Registered nurses	29–1141	$93,973	36%
Dietitians and nutritionists	29–1031	$71,800	27%
Clinical laboratory technologists and technicians	29–2010	$62,440	25%
Recreational therapists	29–1125	$60,857	34%
Exercise physiologists	29–1128	$57,497	19%
Master's degree			
Nurse anesthetists	29–1151	$217,223	38%
Physician assistants	29–1071	$130,887	38%
Nurse practitioners	29–1171	$128,390	35%
Nurse midwives	29–1161	$127,377	36%
Genetic counselors	29–9092	$98,047	56%
Occupational therapists	29–1122	$95,943	23%
Speech-language pathologists	29–1127	$92,643	25%
Orthotists and prosthetists	29–2091	$80,023	14%
Athletic trainers	29–9091	$61,073	36%
Doctoral or professional degree			
Family medicine physicians	29–1215 a	$240,693	NA
Dentists, general	29–1021	$186,713	13%
Podiatrists	29–1081	$161,483	20%
Optometrists	29–1041	$139,013	25%
Veterinarians	29–1131	$135,227	41%
Pharmacists	29–1051	$133,803	15%
Physical therapists	29–1123	$100,267	22%
Audiologists	29–1181	$92,477	24%
Chiropractors	29–1011	$88,437	12%
All U.S. occupations	NA	$65,097	40%

Abbreviations: BLS, Bureau of Labor Statistics; SOC, Standard Occupational Code.

a NA = 2012 to 2014 data not available for comparison.

Within the doctoral and professional degree cohort, audiology ranks eighth of nine occupations by mean wage, clustering more closely with master's-level occupations such as occupational therapy and speech-language pathology than with doctoral-level peers such as optometrists, physical therapists, or pharmacists. As shown in Table 1 , audiologists wage growth from 2012–2014 to 2022–2024 was 24%; 8 percentage points below the healthcare benchmark of 32% and 16 percentage points below the growth rate for all U.S. occupations. These are not the outcomes of a profession undervalued by accident. They are consistent with the predictable labor market expression of constrained pricing authority, limited-service differentiation, and revenue structures that weaken the link between clinical expertise and independently captured income. 11 12

Workforce structure compounds these earnings constraints. The predominance of salaried and hybrid employment models in audiology—common in hospital systems, physician-owned practices, and large hearing care organizations—limits long-term income growth by capping productivity-based upside and weakening the alignment between experience, specialization, and compensation. 12 Independent practice ownership, which in optometry 13 and veterinary medicine 14 functions as the primary mechanism for converting product-linked care into scalable professional revenue, remains the exception rather than the norm in audiology. Additionally, the absence of reimbursement mechanisms that support delegation, supervision, or tiered service delivery reinforces reliance on one-to-one clinical encounters as the fundamental unit of production. Therapy-based professions have developed scalable delivery through aide utilization, group treatment models, and supervised care pathways—structures that allow professional labor to extend beyond individual clinician time. Audiology has not consistently developed analogous models, in part because the reimbursement architecture does not support them. The result is a workforce whose productivity ceiling is structurally bounded by individual clinician availability, limiting both revenue generation and the capacity to meet growing population needs.

These labor market outcomes are not driven by insufficient demand for hearing care. Prevalence of hearing loss is well-documented and growing, and evidence linking untreated hearing impairment to significant health consequences continues to accumulate. 15 The constraint is not population need—it is institutional demand for audiologic services as currently structured within payment and delivery systems. When clinical labor cannot be reliably translated into independently reimbursable activity, and when revenue is mediated through referral requirements, device distribution channels, and benefit structures that capture value upstream of the clinician, workforce growth and earnings stability are predictably limited. Addressing these outcomes requires changes not at the level of individual clinician productivity or professional advocacy alone, but at the level of the institutional architecture—taxonomy, reimbursement, and service delivery models—that determine how audiologic labor is recognized and valued. That is the focus of the section that follows.

## Conclusion

This manuscript has examined audiology's economic position through the integrated lens of professional taxonomy, revenue generation, and labor market outcomes—not as independent domains, but as an interdependent system whose misalignment produces the earnings patterns and workforce dynamics documented throughout this special issue. The central argument is structural: audiology's constrained economic authority is not the consequence of insufficient training, limited clinical scope, or lack of patient demand. It is the predictable outcome of an institutional architecture in which how the profession is classified, how it generates revenue, and how its labor is deployed have not evolved in coordination with one another.

The mechanisms underlying this misalignment are specific and documentable. At the level of taxonomy, audiology's designation as a supplier of diagnostic services under Section 1861 of the Social Security Act restricts independent billing authority, excludes access to E&M reimbursement for clinical activities already routinely performed, and reinforces referral-dependent care models that decouple population-level demand for hearing care from institutional demand for audiologic services. 4 At the level of revenue generation, the profession occupies a hybrid position—simultaneously medical, retail, and therapeutic—without full economic recognition in any of those frameworks, and without the practice ownership patterns and pricing authority that allow structurally analogous professions to convert product-linked care into scalable professional revenue. 5 6 7 At the level of labor markets, these constraints produce the outcomes the companion wage analysis documents in detail: earnings compression within the doctoral degree cohort, wage convergence with a master's-level profession despite a full educational degree of separation, growth trajectories below both healthcare and national benchmarks, and a workforce productivity ceiling structurally bounded by one-to-one service delivery. 10

Within the context of this special issue, this structural account provides the explanatory layer that wage benchmarking and workforce supply analyses alone cannot. Earnings data describe where audiology sits within the healthcare labor market; this analysis characterizes why it sits there, and why it has remained there despite expanding training requirements and growing population need. The observed patterns are not anomalies to be explained away—they are the coherent, predictable outputs of a system in which taxonomy has not conferred the reimbursement authority that training warrants, revenue models have not consistently translated clinical expertise into independently valued services, and labor deployment has remained constrained by the delivery structures those revenue models produce. Understanding this architecture is a prerequisite for interpreting the profession's current challenges accurately—and for framing the evaluative questions that the analyses which follow are positioned to address.

This structural account is necessarily prior to—and distinct from—evaluation of whether specific interventions can produce alignment, or at what pace. The architecture described here sets the terms for that evaluation: what would need to change, in what domains, and why isolated reforms are unlikely to be sufficient. That analysis, drawing on the structural account developed here, is the work of the companion health economic evaluation (see "Professional Status of Audiology in U.S. Healthcare" in this issue).
